# Interventions to Enable or Improve Evidence-Informed Decision Making in Public Health and Preventive Medicine: A Scoping Review

**DOI:** 10.1016/j.focus.2025.100473

**Published:** 2025-12-11

**Authors:** Emily Groot, Jessica Pelley, Anne-Marie Boylan, David Nunan

**Affiliations:** 1 Section of Public Health and Preventive Medicine, Clinical Sciences Division, NOSM University, Sudbury, Canada; 2 Nuffield Department of Primary Care Health Sciences, University of Oxford, Oxford, United Kingdom; 3 Department of Family Medicine, Queen’s University, Kingston, Canada

**Keywords:** Public health, education, public health professional, medical, evidence-based practice, clinical decision making

## Abstract

**Introduction:**

Public health and preventive medicine is the branch of medicine that implements population-level health interventions. Public health and preventive medicine specialists practice at the intersection of medicine and public health. These 2 disciplines approach the application of evidence to decision making differently, and it is unclear how public health and preventive medicine specialists are trained to navigate between these approaches. To better understand the interventions available to support public health and preventive medicine physicians in making evidence-informed decisions in this space, the authors conducted a scoping review to summarize planned or implemented interventions that support or are intended to support evidence-informed decision making in public health and preventive medicine practice.

**Methods:**

In March and April 2025, the authors searched multiple databases and prespecified websites to identify published and unpublished academic and gray literature reports published in English between 1992 and March 2025 that described interventions intended to enable or improve evidence-informed decision making in public health and preventive medicine practice. Data extracted included, where reported, publication date, authors, title, publication source, actual or intended participants, participant characteristics, intervention characteristics, funding sources, and intervention outcome.

**Results:**

The authors identified 246 records, 234 through database and website searches and 12 through citation searches, of which 9 were duplicates. Ultimately, the authors included 17 reports that described 7 unique interventions in this scoping review. The majority (11 of 17) of reports described interventions that took place in the U.S., and most (9 of 17) reports described an iteration of the Evidence-Based Public Health Course. All interventions reported success, most often in terms of positive participant reaction, self-reported participant learning, or participant behaviour change. One intervention, an organizational strategic plan that supported evidence-informed decision-making capacity, reported organizational- and population-level impacts.

**Discussion:**

This scoping review did not identify any interventions specifically targeting evidence-informed decision making in public health and preventive medicine, but there was some indication that there are differences between the types of evidence that physicians value most and those by other public health professionals. The components of evidence-informed decision making in public health implemented or taught in the interventions identified in this scoping review differ from the components of evidence-based medicine taught in undergraduate and postgraduate medical education, which has implications for physicians working in public health.

## Highlights

• Public health and preventive medicine (PHPM) physicians practice at the intersection of medicine and public health.
• Medicine and public health approach the application of evidence to decision making differently.
• It is unclear how PHPM specialists are trained to navigate between these approaches.
• The authors conducted a scoping review to delineate this gap.
• No interventions specifically targeting PHPM have been reported in the literature.

## INTRODUCTION

Public health and preventive medicine (PHPM) is the branch of medicine that plans, implements, and evaluates population-level health interventions.^1^^,^^2^ Historically, PHPM was primarily concerned with communicable disease control.^3^ Today, the scope of PHPM also includes prevention of noncommunicable disease and promotion of well-being.^4^

PHPM exists in the liminal, overlapping space between medicine and public health.^2^^,^^5^^,^^6^ The disciplines of medicine and public health approach the application of evidence to decision making differently.^7^, ^8^, ^9^, ^10^, ^11^, ^12^ Evidence-based medicine (EBM) is an approach to teaching and practicing medicine that “de-emphasizes intuition, unsystematic clinical experience, and pathophysiologic rationale as sufficient grounds for clinical decision making and stresses the examination evidence from clinical research.”^13^ EBM was introduced in the early 1990s at McMaster University in Ontario, Canada, initially as a mechanism to incorporate the rapid increase in RCT data into clinical practice.^13^ EBM’s focus has evolved over time, but at its core, it is an approach to incorporating best evidence in making medical decisions with individual patients.^13^, ^14^, ^15^ For example, should a patient living with HIV be diagnosed with a latent tuberculosis infection, a physician applying an EBM approach would clarify the question, identify the best available evidence, critically appraise the evidence, apply the evidence, and evaluate the application,^16^ in line with the patient’s values and preferences.^13^ Together, the physician and the patient would weigh the benefits and risks of different latent tuberculosis infection treatment regimens and their potential to interfere with HIV medications.

Public health, on the other hand, focuses on population-level interventions and decisions. For example, should a public health agency receive funding to carry out a respiratory health program, an agency applying the GRADE Evidence to Decision approach would consider the benefits and harms of different options—perhaps tuberculosis prevention, smoking cessation, or occupational health hazard control, the balance of effects, the resources required, cost effectiveness, equity, acceptability, and feasibility.^17^ In other words, in public health decision making, best available evidence refers not only to research evidence but also public opinion, partner preference, population burden, health equity impact, funding and costs, human resources, and other complex and intersecting factors.^17^ As a result, EBM approaches focused on individual patients can be difficult to translate to public health practice. Whereas some authors use the term evidence-based public health,^18^^,^^19^ others refer to the practice of judicial review^20^^,^^21^ or evidence-informed decision making (EIDM) to signal that, although evidence of intervention efficacy or effectiveness is considered if available, it is only 1 element in the public health decision-making process.^21^^,^^22^

It is unclear how (or whether) PHPM specialists are trained to navigate between these 2 approaches to decision making, especially when managing the tensions that can arise when what is best for an individual patient is not best for a community or vice versa—for example, requiring an individual with a communicable disease to avoid contact with others in the community.^23^^,^^24^ To understand which interventions to undertake, PHPM physicians lead health surveillance and population health assessments.^7^ To support these activities, PHPM residency training includes epidemiology, biostatistics, and program implementation and evaluation, resulting in expertise in the interpretation and application of population-level health evidence.^1^ Although there are a number of reviews that describe interventions meant to enable or improve EIDM in the context of an individual patient–provider relationship^25^, ^26^, ^27^ or nonphysician public health practice,^22^^,^^28^ the authors are not aware of reviews specific to PHPM.

To better delineate this gap, the authors conducted a scoping review to summarize planned or implemented interventions that support or are intended to support EIDM in PHPM practice or public health practice including PHPM, regardless of efficacy or effectiveness. Specifically, in this review, intervention referred to any structured activity, program, or strategy designed to enhance knowledge, skills, or behaviour; EIDM referred to the systematic process of integrating the best available research and epidemiologic evidence with clinical expertise, community context, and stakeholder values^22^^,^^29^; and PHPM practice referred to the intersection of public health and medicine.^2^^,^^5^

## METHODS

This scoping review sought to address the question, “What interventions intended to enable or improve EIDM in PHPM practice have been reported in the academic and gray literature?” The review was conducted in accordance with the Joanna Briggs Institute methodology for scoping reviews^30^^,^^31^ and the PRISMA 2020 statement,^32^ insofar as the latter applies to scoping reviews. The review was registered with the Center for Open Science’s Open Science Framework on March 15, 2025, using the Generalized Systematic Review Registration Form.^33^, ^34^, ^35^ Deviations from this protocol have been reported and justified below.

The population of interest for this scoping review was PHPM physicians. The authors decided in advance that if the search did not identify interventions specific to PHPM physicians, interventions for the population of public health professionals, including PHPM physicians, would be included. The concept of interest for this scoping review was planned or implemented interventions that support EIDM in PHPM practice or public health practice that includes PHPM, including interventions that occurred during PHPM residency training. Finally, the context for this scoping review was global, and interventions from around the world were considered. The scoping review considered any type of report, including gray literature reports, studies, theses, and conference abstracts of any design, including but not limited to experimental and quasiexperimental study designs, observational studies, descriptive studies, qualitative studies, opinion papers, program and curriculum plans, policies and policy analyses, and reviews (systematic reviews, scoping reviews, critical reviews).

This review excluded interventions aimed solely at nonphysician public health practice (e.g., public health nurses, public health inspectors, health promoters) as well as interventions aimed at medical student education or non-PHPM physician practice (e.g., general practitioners, family physicians, internists). Owing to the language limitations of the reviewers, only reports published in English were included. This review also excluded reports published prior to 1992, the year EBM was first defined by Guyatt and colleagues.^13^

The multistep search strategy aimed to locate both published and unpublished academic and gray literature reports. The authors conducted an initial, limited Google Scholar search to identify reports concerning interventions to enable or improve EIDM in PHPM. The text of the titles and abstracts of relevant reports as well as the index terms from that initial search were used to develop a full-search strategy for the databases and information sources described in Table 1. The search was conducted between March 18, 2025 and April 13, 2025.

**Table 1:** Description of Databases and Information Sources

Database or information source	Description
Ovid MEDLINE	Database of academic life sciences and biomedical journals
MedEdMENTOR Paper Database	Database of medical education papers
Overton	Index of policy documents
ProQuest Educational Resources Information Center	Database of academic and gray literature related to education
Grey Matters	Database of health-related gray literature
Google Scholar	Web search engine that searches scholarly literature
Prespecified websites	National Collaborating Centre for Methods and Tools, U.S. Community Preventive Services Task Force, National Association of County & City Health Officials, Cochrane Public Health, and Campbell Collaboration

The search strategy, including all identified keywords and index terms, was adapted for each included database or information source. The key words, MeSH (Medical Subject Heading) terms, and search strings are listed in Appendix A Tables 1 and 2 (available online), including the multiple synonyms used in different countries to refer to the specialty of PHPM.^6^^,^^36^, ^37^, ^38^, ^39^ The search strings specific to each database in Table 1 are listed in Appendix A Table 3 (available online). The authors screened the reference lists of all included sources for additional reports using the key words listed in Appendix Table 1 (available online). The authors also screened forward citations of all included reports of evidence using Web of Science for the same key words.

After the search, all identified citations were collated and documented in a shared Microsoft 365 Excel spreadsheet, and duplicates were removed. In the first round of screening, 2 reviewers (EG and JP) independently screened titles against the inclusion criteria using the questions listed in Table 2.

**Table 2:** Screening Questions

1	Is the report written in English?
2	Was the report published in or after 1992?
3	Does the report (appear to) describe an intervention (any structured activity, program, or strategy designed to enhance knowledge, skills, or behaviour)?
4	Is the intervention aimed (or appears to be aimed) to enable or improve evidence-informed decision-making (systematic process of integrating the best available research and epidemiological evidence with clinical expertise, community context, and stakeholder values)?
5	Is the intervention aimed (or does it appear to be aimed): at PHPM residents or specialists specifically? at public health practitioners, including PHPM residents or specialists?

PHPM, public health and preventive medicine.

In the second round of screening, the reviewers (EG and JP) screened abstracts, again using the screening questions listed in Table 2. Potentially relevant reports were retrieved in full, and their citation details were imported into Mendeley Desktop, Version 1.19.8 (2008). Deviating from the pre-established protocol, the reports were shared between reviewers in a shared Microsoft 365 folder instead of a synchronized Mendeley web account owing to ease of use. After the abstract screening, full texts were assessed in detail against the inclusion criteria by the same 2 reviewers. Reasons for exclusion of full-text reports were recorded. Disagreements between the reviewers were resolved through discussion.

EG and JP independently extracted data from the included reports using the data extraction tool in Appendix Table 4 (available online). EG piloted the data extraction tool on 3 randomly selected reports. After the pilot, the data extraction tool was modified to include funding sources. The data extracted included, where reported, publication date, authors, title, publication source, actual or intended participants, participant characteristics, intervention characteristics, funding sources, and intervention outcome.

## RESULTS

The authors identified 246 records, 234 through database and register searches and 12 through citation searches, of which 9 were duplicates. The majority of results (152 of 187) were identified through bibliographic databases of academic journals (Ovid MEDLINE, MedEdMentor, and Google Scholar). The remaining results were identified through the gray literature bibliographic databases and prespecified websites listed in Table 1. Because the reports identified in the first round of citation searches only described interventions already included in the scoping review, the authors did not perform a second round of citation searches (i.e., reports included as a result of the first citation search did not undergo further citation search).

**Figure 1:**
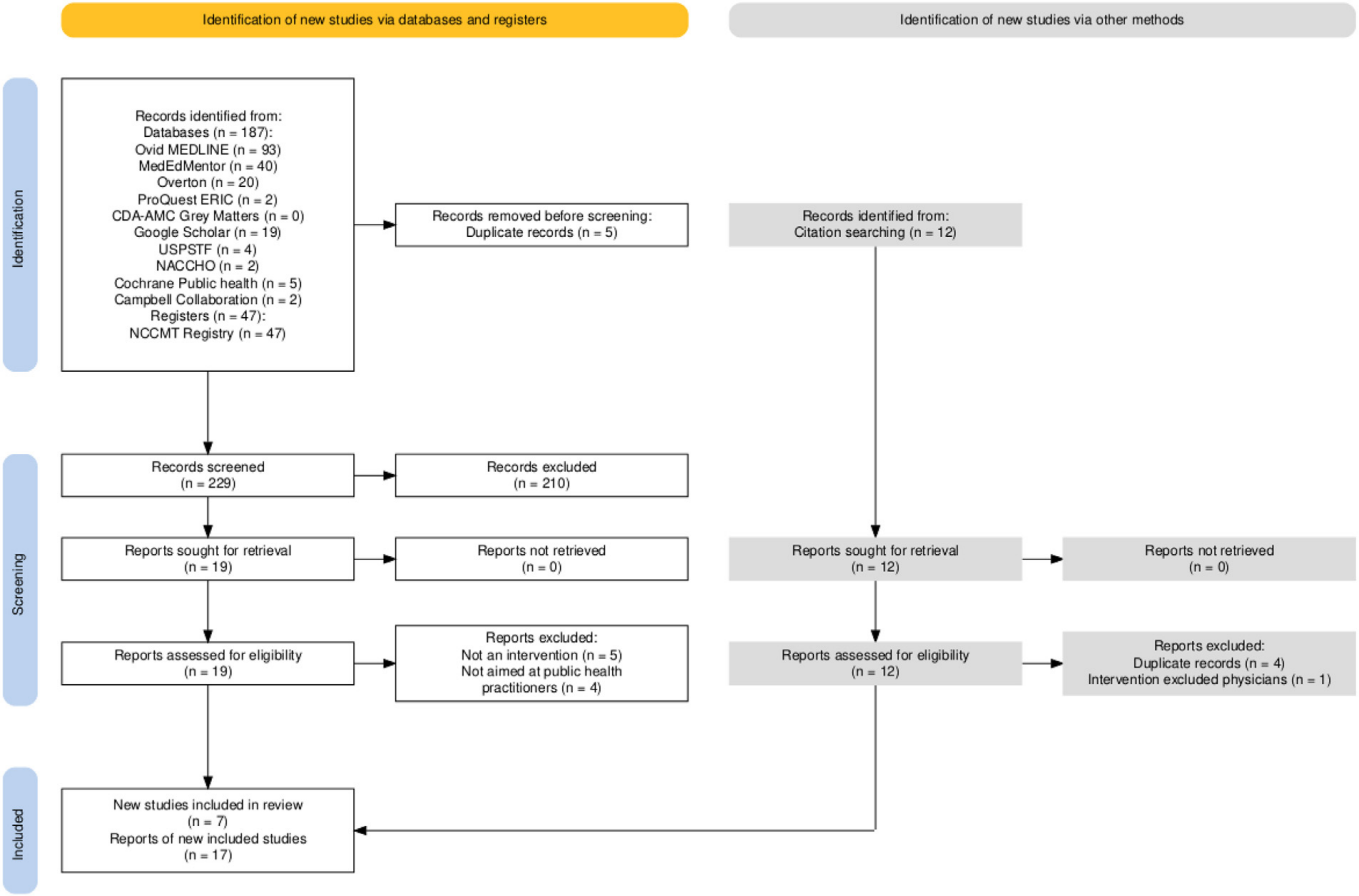
PRISMA flow diagram. *Note*: Image was generated using the Shiny app for producing PRISMA 2020–compliant flow diagrams.^40^ CDA-AMC, Canada’s Drug Agency-L’Agence des medicaments due Canada; NACCHO, National Association of County and City Health Officials; NCCMT, National Collaborating Centre for Methods and Tools; USPSTF, U.S. Preventive Services Task Force.

After review of the remaining 237 records, 27 full-text reports were retrieved and reviewed. Ten full-text reports were excluded, either because the report described an initiative or project that was not an intervention (e.g., a framework with no evidence of planned or actual implementation) or because the report described an intervention that specifically excluded PHPM physicians or residents. Ultimately, the authors included 17 reports describing 7 unique interventions in this scoping review. The results are presented in Figure 1^40^—PRISMA 2020 flow diagram for new systematic reviews.^32^ Appendix B (available online) summarizes the reports and interventions included in the scoping review as well as lists excluded studies with the reasons for exclusion.

The majority (11 of 17) of reports described interventions that took place in the U.S. Some interventions took place in multiple countries. In addition to the U.S., the authors identified interventions implemented in Australia, Brazil, Burkina Faso, Canada, Chile, Colombia, Georgia, Guatemala, India, Liberia, Lithuania, Malawi, Mexico, Russia, and Saudi Arabia.

Most (9 of 17) reports described an iteration of the Evidence-Based Public Health (EBPH) Course.^41^, ^42^, ^43^, ^44^, ^45^, ^46^, ^47^, ^48^, ^49^ The EBPH Course was developed in 1997 by the St. Louis University School of Public Health in collaboration with the Missouri Department of Health and Senior Services.^46^ The course is offered in 2.5 to 4-day formats.^42^^,^^47^ The objective of the EBPH Course is to equip public health professionals with the skills and knowledge to make evidence-informed decisions, with the ultimate goal of preventing chronic disease.^41^^,^^45^^,^^49^ From 1997 to 2016, the EBPH Course consisted of 9 modules: an introduction and overview, assessing and engaging communities, quantifying the issue, developing a concise statement of the issue, searching and summarizing the scientific literature, developing and prioritizing intervention options, developing an action plan and building a logic model, understanding economic evaluation, and evaluating the program or policy.^46^^,^^49^ A tenth module covering communicating and disseminating evidence to policymakers was added in 2017.^48^^,^^49^ The course has been delivered in all 50 U.S. states, 2 U.S. territories, and multiple countries,^49^ by both local public health faculty^46^ and a train-the-trainer approach.^47^ It has been adapted for specific contexts, for example, in the New York State Department of Health^44^ and into an existing Pan American Health Organization/WHO capacity building program.^45^

An additional 2 reports describe the Public Health Leadership and Implementation Academy for Non-Communicable Diseases (PH-LEADER for NCDs), a year-long program that includes and expands upon components of the EBPH Course and also includes a mentorship period to support the initiation of a project.^50^^,^^51^ Similar to the EBPH Course, the objective of the PH-LEADER for NCDs program is to support public health professionals in developing skills and knowledge to effectively use evidence, with the ultimate goal of reducing the burden of noncommunicable diseases in low- and middle-income countries.^50^^,^^51^

Of the remaining 6 reports, 2 describe the implementation and outcomes of an organizational strategic plan intended to improve EIDM in local public health practice,^52^^,^^53^ and 1 report describes each of the application of an evidence-informed public health framework,^54^ tailored evidence-informed public health short courses,^55^ a public health preventive medicine residency program curriculum,^56^ and interventions delivered by a knowledge broker at local public health agencies.^57^ One of the interventions delivered by the knowledge broker in Yost et al.^57^ includes the application of the same evidence-informed public health framework described by Martin and colleagues.^54^

Two of the interventions, the EBPH Course and the PH-LEADER for NCDs, focused explicitly on EIDM in chronic disease prevention. The other interventions were either cross cutting or did not specify an area of public health focus. Although one report indicated that an EBPH Course focused on infectious disease was planned but not yet implemented,^48^ the authors did not identify any interventions focused explicitly on EIDM in communicable disease control, environmental health protection, or emergency management.

All of the interventions were reported to be successful, with success defined differently across reports. Most interventions reported success in terms of participant reaction, such as satisfaction with the intervention or improved self-confidence in EIDM,^44^^,^^55^^,^^56^ self-reported participant learning,^45^^,^^46^^,^^57^ or participant behaviour changes such as publications or applying new skills in their work.^41^^,^^42^^,^^50^^,^^51^^,^^54^ Only 1 intervention, an organizational strategic plan that supported EIDM capacity, reported organizational- and population-level impacts: the adoption of multiple new evidence-informed programs and the cessation of programs that were found not to be supported by evidence.^52^^,^^53^

## DISCUSSION

This scoping review did not identify any interventions specifically targeting EIDM in PHPM. Instead, this review summarizes interventions aimed at public health practitioners, including physicians. Only 1 intervention, the residency program curriculum, was explicitly designed for and delivered to PHPM physicians. Although 1 of the objectives of this curriculum was to “address the increasing demand for evidence-based IM [integrative medicine] by training physicians to implement cost-effective primary and secondary prevention services and programs,” the primary goal was to increase physician skill and application of complementary and alternative medicine.^56^ Because the evidence base for some complementary and alternative medicine interventions is contended,^58^ components of this curriculum are unlikely transferrable to other EIDM contexts.

Although this review did not identify any other interventions specifically designed for the medical specialty of PHPM, there was some indication that there are differences in the types of evidence that physicians value compared with that of other public health professionals. Martin et al.^54^ describe that the medical health officers in their study tended to value epidemiologic data and research evidence more highly, whereas other public health practitioners preferentially valued experiential evidence.

Many of the reports identified organizational leadership and, sometimes specifically physician leadership, as a key component in initiating and maintaining EIDM in public health writ large. Some of the success of reorienting the Peel Region Public Health Department to EIDM was attributed to physician leadership: Peirson and colleagues^53^ noted that “Staff thought without the [medical officer of health]’s vision and commitment, this initiative would not have started and they believed his continued involvement will be a key factor contributing to its success.” That the agency assigned 0.5 full-time equivalents of an associate medical officer of health’s position to supporting EIDM was also perceived to be critical to the intervention’s success.^53^ The PH-LEADER for NCDs program was in part developed in recognition that disseminating and implementing evidence require effective organizational leadership.^50^^,^^51^ As summarized in Table 3,^16^^,^^48^^,^^54^^,^^55^^,^^57^ the components of EIDM in public health implemented or taught in the interventions identified in this scoping review differ from the components of EBM.

**Table 3:** Comparison of Components of Evidence-Based Medicine With the Evidence-Informed Decision-Making Frameworks Identified in the Scoping Review

Evidence-based medicine^16^	NCCMT EIPH Framework^54^^,^^57^	EIPH Short Course^55^	EBPH Course^48^
Clarify the question Identify the best available evidence Critically appraise the evidence Apply the evidence Evaluate the application	Define the problem Search for the best available evidence Appraise the quality of the evidence Synthesize/interpret the evidence Adapt to the local context Implement if appropriate Evaluate the implementation	Ask an answerable question Find evidence to answer the question Assess the trustworthiness of the evidence Integrate the evidence with practitioner expertise and values of the population and evaluation Evaluate to generate evidence to contribute back to the process	Develop an initial statement of the issue Quantify the issue Search the scientific literature and organize information Develop and prioritize program options Develop an action plan and implement interventions Evaluate the program or policy Disseminate or discontinue

EBPH, Evidence-Based Public Health; EIPH, Evidence-Informed Public Health; NCCMT, National Collaborating Centre for Methods and Tools.

In contrast to EBM, EIDM decision making in public health is positioned at a team or agency level, requiring organizational resources such as librarians, epidemiologists, and policy analysts, rather than at the level of an individual practitioner. Also in contrast to EBM, which traditionally replies on the best available syntheses of analytical studies,^13^^,^^25^^,^^59^ but consistent with the GRADE Evidence-to-Decision framework,^17^ the interventions identified in this review encourage public health practitioners to consider a broader range of evidence in decision making—including indicators of population health, economic analyses, community preference, and program evaluation, in addition to evidence of intervention effectiveness. Although not explicitly discussed by any of the reports in this review, this appears to reflect a difference in paradigm between the 2 disciplines, with EBM aligning more closely with a positivist ontology and EIDM in public health aligning more closely with a realist ontology.

Given that (1) leadership is an important component in EIDM in public health, (2) physicians often serve as leaders in public health agencies, and (3) the approach to EIDM is taught differently in public health than in medicine, it is particularly surprising that this review did not identify any interventions designed to enable or improve EIDM specifically in PHPM practice. This gap may have particular impact on low- and middle-income regions, where capacity and funding EIDM are already more limited owing to inequitable distribution of global resources.^22^ Addressing this gap will prepare PHPM specialists to address existing population health needs as well as emerging challenges such as climate change,^60^ mis and disinformation,^61^ future pandemic preparedness,^29^ and growing health inequities.^62^

### Limitations

To the authors’ knowledge, this is the first scoping review that summarizes planned or implemented interventions that support or are intended to support EIDM in public health practice, including PHPM. The search strategy was comprehensive, including both gray and academic literature. The scoping review methodology is particularly appropriate for summarizing the breadth of existing literature on a topic and identifying gaps in that literature.^63^

Although this review has considerable strengths, it also has some limitations. Consistent with scoping review methodology,^30^^,^^63^ this review did not assess the risk of bias or critically appraise the quality of the evidence. In other words, this review summarizes what has been done but not how well it worked. The search was restricted to English-language reports. Furthermore, the lead author is a Canada-based PHPM physician who identified the nontraditional databases and registry on the basis of her clinical experience (i.e., CMA-AMC Grey Matters, NCCMT Registry, USPSTF, NACCHO, Cochrane Public Health, Campbell Collaboration), only 2 of which are based outside North America. As a result, relevant non-English reports and interventions developed or implemented outside Canada or the U.S. may have been missed in the search. In addition, interventions that are not referenced by public-facing documents, such as residency curriculum documents or internal public health agency training, would have been missed in the search.

## CONCLUSIONS

The authors set out to summarize planned or implemented interventions that support or are intended to support EIDM in PHPM practice. The authors were unable to identify any interventions specifically targeting PHPM physicians. As a result, the authors summarized planned or implemented interventions that support or are intended to support EIDM in public health practice, including PHPM physician practice.

This scoping review highlights a significant gap in the interventions available to enable or improve EIDM in public health practice: no interventions specifically targeting PHPM physicians have been reported in the academic or gray literature. PHPM physicians work in the intersecting space between medicine and public health. The disciplines of medicine and public health have differing approaches to the synthesis and application of evidence to decision making. The authors suggest that future research should explore how to integrate these approaches within PHPM practice and how to most effectively support EIDM at the intersection of medicine and public health, such as a curriculum or training program specific to PHPM physicians or residents.

## CRediT authorship contribution statement

**Emily Groot:** Conceptualization, Methodology, Investigation, Writing – original draft, Project administration. **Jessica Pelley:** Investigation, Writing – review & editing. **Anne-Marie Boylan:** Writing – review & editing, Supervision. **David Nunan:** Writing – review & editing, Supervision.

## Appendix Supplementary materials

Image, application 1

Image, application 2

## Footnotes

Supplementary material associated with this article can be found in the online version at doi:10.1016/j.focus.2025.100473.
