# Supplementary material for: Interventions to Enable or Improve Evidence-Informed Decision Making in Public Health and Preventive Medicine: A Scoping Review
Source: AJPM Focus. 2025 Dec 11;5(3):100473. doi: 10.1016/j.focus.2025.100473 (PMC13049646; doi:10.1016/j.focus.2025.100473)
Supplement: Supplementary file 1 [file mmc1.doc]

# Interventions to enable or improve evidence-informed decision-making in public health and preventive medicine: A scoping review

## Appendix A: Search strategy

### Table 1: Key words

| **Key word** | **Author definition** |
| --- | --- |
| Public health and preventive medicine | Term used in some countries, including the United Kingdom and Canada after 2011, to describe the branch of medicine focused on population-level health interventions (36) |
| Community medicine | Term used in some countries, including India and Canada prior to 2011, to describe the branch of medicine focused on population-level health interventions (6,37) |
| Public health physician OR public health doctor | Physician who works with a public health agency; may or may not be a PHPM specialist |
| Public health | The organized efforts of society to prevent disease and promote health (38) |
| Hygiene and preventive medicine | Term used in Italy to describe the branch of medicine focused on population-level health interventions (36) |
| Preventive medicine | Term used in the United States to describe the branch of medicine focused on preventive care, which can include population-level health interventions (36,39) |
| Public health and social medicine | Term used in France to describe the branch of medicine focused on population-level health interventions (36) |
| Evidence-based medicine | The process of asking a clinical question, identifying the best available evidence, critically appraising the evidence, applying the evidence to a clinical scenario, and evaluating the outcome (16) |
| Evidence-informed decision-making | The process of incorporating the best available evidence into decision-making, while still recognizing evidence is only one element in the decision-making process (22) |
| Evidence-informed practice | The ongoing practice of evidence-informed decision-making (22) |
| Evidence-based OR evidence-informed | An outcome or process that is based in or informed by evidence |

### Table 2. Medical subject heading (MeSH) terms

| **MeSH term** | **Definition** | **Included MeSH sub-terms** |
| --- | --- | --- |
| Education, Medical | Use for general articles concerning medical education. | Education, Medical, Continuing  Education, Medical, Graduate  Education, Medical, Undergraduate  Teaching Rounds |
| Education, Public Health Professional | Education and training in PUBLIC HEALTH for the practice of the profession. | None |
| Public Health (under Population Characteristics) | Branch of medicine concerned with the prevention and control of disease and disability, and the promotion of physical and mental health of the population on the international, national, state, or municipal level. | None |
| Public Health (under Environment and Public Health) | Branch of medicine concerned with the prevention and control of disease and disability, and the promotion of physical and mental health of the population on the international, national, state, or municipal level. | Accidents  Consumer Product Safety  Disease Eradication  Disease Outbreaks  Disease Transmission, Infectious  Drug Contamination  Emergencies  Endemic Diseases  Environmental Medicine  Environmental Microbiology  Environmental Pollution  Epidemiologic Factors  Epidemiologic Measurements  Epidemiologic Methods  Equipment Contamination  Equipment Reuse  Food Quality  Health Transition  Hygiene  Legal Epidemiology  Patient Harm  Public Health Practice  Radiologic Health  Sanitation |
| Public Health (under Health Occupations) | Branch of medicine concerned with the prevention and control of disease and disability, and the promotion of physical and mental health of the population on the international, national, state, or municipal level. | Epidemiology  Preventive Medicine |
| Evidence-Based Practice | A way of providing health care that is guided by a thoughtful integration of the best available scientific knowledge with clinical expertise. This approach allows the practitioner to critically assess research data, clinical guidelines, and other information resources in order to correctly identify the clinical problem, apply the most high-quality intervention, and re-evaluate the outcome for future improvement. | Evidence-Based Dentistry  Evidence-Based Medicine  Evidence-Based Nursing  Evidence-Based Pharmacy Practice |
| Social Medicine | A branch of medicine concerned with the role of socio-environmental factors in the occurrence, prevention and treatment of disease. | None |
| Clinical Decision-Making | Process of formulating a diagnosis based on medical history and physical or mental examinations, and/or choosing an appropriate intervention. | Clinical Reasoning |
| Public Health Practice | The activities and endeavors of the public health services in a community on any level. | Communicable Disease Control  Decontamination  Environmental Monitoring  Mass Screening  Medical Countermeasures  Population Surveillance  Primary Prevention  Quaternary Prevention  Secondary Prevention  Tertiary Prevention |

### Table 3. Search strings, date, and results (n)

| **Database** | **Ovid MEDLINE** |
| --- | --- |
| **Date** | 18-Mar-25 |
| **Search string** | ((public health and preventive medicine).mp. OR (Social Medicine/) OR (exp Public Health Practice/) OR (public health).mp OR (Public Health/)) AND ((exp Education, Medical/) OR (Education, Public Health Professional)) AND ((evidence-based medicine.mp OR Evidence-Based Medicine/) OR (*Evidence-Based Practice/) OR (clinical decision-making/)) limit 1 to (english language and yr="1992-current") |
| **Results (n)** | 93 |
| **Database** | **MedEdMentor** |
| **Date** | 18-Mar-25 |
| **Search string** | public health preventive medicine evidence-informed decision-making |
| **Results (n)** | 40 |
| **Database** | **Overton: Search policy documents** |
| **Date** | 19-Mar-25 |
| **Search string** | "public health" AND "evidence-informed" |
| **Results (description)** | 20,085 > sorted by relevance > First 20 search results included |
| **Results (n)** | 20 |
| **Database** | **ProQuest Eric** |
| **Date** | 19-Mar-25 |
| **Search string** | (public health) AND evidence Date: After 1992  Document type Article, Bibliography, Book, Conference Proceeding, Directory, Dissertation/Thesis, Editorial, General Information, General_Information, Government & Official Document, Instructional, Instructional Material/Guideline, Journal Article, Reference Document, Report, Research_Bulletins, Review, Speech/Lecture, Statistics, Statistics/Data Report, Supporting_Materials, Teaching_Material, Tests/Questionnaires, Translation Language English Education level Adult education Target audience Policymakers, Practitioners, Researchers, Teachers |
| **Results (n)** | 2 |
| **Database** | **CDA-AMC Grey Matters (https://greymatters.cda-amc.ca/)** |
| **Date** | 19-Mar-25 |
| **Search string** | language:(English) "public health" evidence |
| **Results (description)** | n/a |
| **Results (n)** | 0 |
| **Database** | **Google Scholar** |
| **Date** | 19-Mar-25 |
| **Search string** | "public health and preventive medicine" "evidence-informed" Date range: 1992 - |
| **Results (description)** | "About 430 results" > First 2 pages (19) results included |
| **Results (n)** | 19 |
| **Comments** | Search conducted in Sudbury, Ontario, Canada at 3:47 PM Eastern time. |
| **Database** | **NCCMT Registry of Methods and Tools for Evidence-Informed Decision Making** |
| **Date** | 22-Mar-25 |
| **Search string** | "public health" |
| **Results (description)** | n/a |
| **Results (n)** | 47 |
| **Database** | **US Preventive Services Task Force website** |
| **Date** | 22-Mar-25 |
| **Search string** | evidence-based public health |
| **Results (description)** | 82 > Hand search > 4 |
| **Results (n)** | 4 |
| **Database** | **NACCHO website (naccho.org)** |
| **Date** | 22-Mar-25 |
| **Search string** | evidence based |
| **Results (description)** | 475 search results + unknown number of Tools (under Resources>Toolbox) > Hand search > 2 |
| **Results (n)** | 2 |
| **Database** | **Cochrane Public Health website (ph.cochrane.org)** |
| **Date** | 23-Mar-25 |
| **Search string** | n/a (hand search only) |
| **Results (description)** | "https://ph.cochrane.org/our-publications" lists 36 resources "that may be of interest" > 5 identified for further review |
| **Results (n)** | 5 |
| **Database** | **Campbell Collaboration website (campbellcollaboration.org)** |
| **Date** | 23-Mar-25 |
| **Search string** | n/a (hand search only) |
| **Results (description)** | Campbell Collaboration > Our work > Knowledge Translation and Implementation (KTI) > Reviews > 7 reviews published > 2 potentially relevant to this project |
| **Results (n)** | 2 |

### Table 4: Data extraction instrument

| **Publication date** | **Authors** | **Title** | **Publication source** | **Participants** | **Participant character-istics** | **Intervention goals and objectives** | **Intervention character-istics** | **Intervention outcomes** | **Author funding sources** |
| --- | --- | --- | --- | --- | --- | --- | --- | --- | --- |
|  |  |  |  |  |  |  |  |  |  |
|  |  |  |  |  |  |  |  |  |  |
|  |  |  |  |  |  |  |  |  |  |
|  |  |  |  |  |  |  |  |  |  |
|  |  |  |  |  |  |  |  |  |  |
