# Supplementary material for: Interventions to Enable or Improve Evidence-Informed Decision Making in Public Health and Preventive Medicine: A Scoping Review
Source: AJPM Focus. 2025 Dec 11;5(3):100473. doi: 10.1016/j.focus.2025.100473 (PMC13049646; doi:10.1016/j.focus.2025.100473)
Supplement: Supplementary file 2 [file mmc2.doc]

# Interventions to enable or improve evidence-informed decision-making in public health and preventive medicine: A scoping review

## Appendix B: Search Results

### Table 1. Included study characteristics

|  | **Authors** | **Year of publication** | **Intervention** | **Study design** | **Participant type** | **Participants (n)** | **Location** |
| --- | --- | --- | --- | --- | --- | --- | --- |
|  | Martin et al. | 2017 | Evidence-Informed Public Health Framework | Secondary analysis of qualitative data | Public health practitioners | 78 interviewees | Canada |
|  | Peirson, Ciliska, & Dobbins | 2012 | Strategic plan | Pre- and post-intervention qualitative interviews | Public health practitioners | 70 key informants | Canada |
|  | Pettman et al. | 2013 | Tailored evidence-informed public health short courses | Pre- and post-intervention survey | Public health practitioners | 45 pre-course, 59 post-course, 38 follow-up survey respondents | Australia |
|  | Brownson et al. | 2017 | Evidence-Based Public Health Course | Randomized controlled trial | Public health practitioners | 6 intervention states, 6 control states | United States |
|  | Dreisinger et al. | 2008 | Evidence-Based Public Health Course | Post-intervention survey | Public health practitioners | 267 course participants, 77 included in analysis | United States |
|  | Ward, Dobbins, & Peirson | 2016 | Strategic plan | Five-year review | Public health practitioners | 650 staff | Canada |
|  | Wells, Benn, & Warber | 2015 | Public Health Preventive Medicine Residency Integrative Medicine Program | Curriculum evaluation | Public health residents | 7 orientation and seminar participants, 4 clinical rotation participants, 3 faculty training participants | United States |
|  | Prevention Research Center in St. Louis | n.d. | Evidence-Based Public Health Course | Randomized controlled trial | Public health practitioners | 6 intervention states, 6 control states | United States |
|  | Maylahn et al. | 2008 | Evidence-Based Public Health Course | Pre- and post-intervention survey and course evaluation | Public health practitioners | 171 training session participants | United States |
|  | Brownson et al. | 2007 | Evidence-Based Public Health Course | Course description and evaluation | Public health practitioners | Not provided | 38 countries |
|  | Yost et al. | 2014 | Interventions delivered by a knowledge broker | Case study | Public health practitioners | 37 interviewees | Canada |
|  | Baker et al. | 2009 | Evidence-Based Public Health Course | Course evaluation | Public health practitioners | 293 course participants, 107 survey respondents | United States |
|  | Yarber et al. | 2015 | Evidence-Based Public Health Course | Program evaluation | Public health practitioners | 317 course participants, 144 survey respondents | United States |
|  | O’Neall & Brownson | 2005 | Evidence-Based Public Health Course | Course evaluation | Public health practitioners | 399 course participants | United States, Russia, Chile, Europe (countries not specified) |
|  | Jacob et al. | 2021 | Evidence-Based Public Health Course | Course evaluation | Public health practitioners | 723 course participants | United States, Other (countries not specified) |
|  | Galaviz et al. | 2016 | Public Health Leadership and Implementation Academy | Impact evaluation | Public health practitioners | 67 course participants | India, Mexico, Brazil, Colombia, Saudi Arabia, Barbados, Guatemala, Malawi, Liberia, United States |
|  | Galaviz et al. | 2019 | Public Health Leadership and Implementation Academy | Course evaluation | Public health practitioners | 90 course participants | India, Mexico, Brazil, Colombia, Saudi Arabia, Barbados, Guatemala, Malawi, Liberia, United States, Burkina Faso |

### Table 2. Included intervention characteristics

|  | **Intervention** | **References** | **Level of intervention** | **Public health topic** | **Description** | **Outcomes** |
| --- | --- | --- | --- | --- | --- | --- |
|  | Evidence-Informed Public Health (EIPH) Framework | - Martin et al., 2017 | Organization | Cross-cutting | The National Collaborating Centres (NCCs) for Public Health were developed to strengthen public health practice in Canada. One of the NCCs, the National Collaborating Centre for Methods and Tools developed the EIPH Framework to guide public health professionals in identifying, synthesizing, and applying high-quality evidence. | Public health practitioners describe regularly using data and evidence in their everyday work. Participants identified that the EIPH Framework lacked direction regarding appraising and synthesizing evidence, especially different types of evidence (e.g., qualitative and quantitative). |
|  | Strategic plan | - Peirson, Ciliska, & Dobbins, 2012 - Ward, Dobbins, & Peirson, 2016 | Organization | Cross-cutting | The 2009-2019 Peel Public Health Strategic Plan identified EIDM as an organizational priority. As a result, the organization introduced EIDM training, tools, knowledge-sharing forums and expanded library and research capacity. | In response to evidence reviews, the agency started 17 programs, stopped 6 programs, changed 20 programs, and did not change 6 programs. |
|  | Tailored evidence-informed public health short courses | - Pettman et al., 2013 | Individual | Cross-cutting | The Public Health Evidence and Knowledge Translation team at the University of Melbourne, affiliated with Cochrane Public Health Group, offers tailored evidence-informed public health short courses. | Participant self-reported confidence in undertaking evidence-informed public health practice increased. |
|  | Evidence-Based Public Health Course | - Brownson et al., 2017 - Dreisinger et al., 2008 - Prevention Research Center in St. Louis, n.d. - Maylahn et al., 2008 - Brownson et al., 2007 - Baker et al., 2009 - Yarber et al., 2015 - O’Neall & Brownson, 2005 - Jacob et al., 2021 | Individual | Chronic disease prevention | The Evidence-Based Public Health Course was developed in 1997 by the St, Louis University School of Public Health in Collaboration with the Missouri Department of Health and Senior Services. It is a 3-, 4-, or 5-day course that includes didactic sessions and case-based learning. It has been expanded and adapted by the US CDC, PAHO/WHO, and the New York State Department of Health. | Regardless of location or modality, participants generally find the course useful. In a randomized controlled trial, where participants attended the course and controls received EIDM resources, participants self-reported greater improvements in EIDM skills like prioritization, community assessment, and evaluation design. |
|  | Public Health Preventive Medicine Residency Integrative Medicine Program | - Wells, Benn, & Warber, 2015 | Individual | Not specified | Residents attended a Graduate Summer Session in Epidemiology (1 month) and reviewed evidence for complementary and alternative medicines. | No EIDM outcomes were specifically described. The addition of integrative medicine to the residency curriculum was mostly positive. |
|  | Interventions delivered by a knowledge broker | - Yost et al., 2014 | Organization | Cross-cutting | A knowledge broker, an individual who works to link evidence users with evidence, worked with three Ontario public health agencies to tailor an intervention to improve EIDM. The interventions variably included: workshops, presentations, mentorship, and one-on-one consultations. | Staff reported the intervention increased their confidence in and engagement with EIDM, and felt the tools were relevant to their future work. Despite support, there was still variable interpretation and application of the tools. |
|  | PH-LEADER for NCDs | - Galaviz et al., 2016 - Galaviz et al., 2019 | Individual | Chronic disease | The PH-LEADER for NCDs is a year-long program with three components: a two-month preparation period, a three-week short course that includes analytic, implementation, and leadership methods and skills, and a mentored project. The average cost was 4,000 USD/participant. | Participants reported a high level of satisfaction with the course, and improvement in leadership skills. As a result of the program, 53 projects were initiated and 20 manuscripts were submitted for publication. |

### Table 3. Reports excluded during full-text review

|  | **Authors** | **Year of publication** | **Title** | **Reason for exclusion** |
| --- | --- | --- | --- | --- |
|  | Jacobs et al. | 2012 | A survey tool for measuring evidence-based decision making capacity in public health agencies | **Not an intervention**: The study described competency gaps but did not propose a specific intervention |
|  | Maddock | 2014 | Academic-practice partnerships for active living: the Healthy Hawaii Initiative | **Not aimed at public health practitioners**: The intervention is aimed at improving the health of Hawaiians through more effective knowledge transfer |
|  | Public Health Agency of Canada | 2011 | Casebook on Evaluation for Learning | **Not an intervention**: The casebook summarizes examples of evaluations with the intention of improving evaluation, but is not a structured activity |
|  | Heenan et al. | 2023 | Combining public health evidence, policy experience and communications expertise to inform preventive health: reflections on a novel method of knowledge synthesis | **Not aimed at public health practitioners**: The target of the intervention is health policymakers in general and is not specific to public health professionals |
|  | Renfrew et al. | 2008 | Developing evidence-based recommendations in public health – incorporating the views of practitioners, service users and user representatives | **Not aimed at public health practitioners**: The intervention is aimed at healthcare providers in general |
|  | Schunemann et al. | 2016 | GRADE Guidelines: 16. GRADE evidence to decision frameworks for tests in clinical practice and public health | **Not an intervention**: Describes a framework, but not its use or application |
|  | Arnold et al. | 2024 | Implementing evidence ecosystems in the public health service: development of a framework for designing tailored training programs | **Not an intervention**: Describes how to develop a course, rather than the implementation of a course |
|  | Wilkinson et al. | 2009 | The role of the information specialist in supporting knowledge transfer: A public health information case study | **Not an intervention**: Describes the creation of research bulletins that might support EIDM on a specific topic, but do not specifically enhance knowledge, skills, or behaviour with respect to EIDM in general |
|  | Meissner et al. | 2013 | The U.S. training institute for dissemination and implementation research in health | **Not aimed at public health practitioners**: The intervention is aimed at health researchers |
|  | Dobbins et al. | 2009 | A randomized controlled trial evaluating the impact of knowledge translation and exchange strategies | **Intervention excluded physicians**: The intervention was targeted at program managers and coordinators and specifically excluded PHPM physicians |
